# HITSZ_CDR: an end-to-end chemical and disease relation extraction system for BioCreative V

**DOI:** 10.1093/database/baw077

**Published:** 2016-06-05

**Authors:** Haodi Li, Buzhou Tang, Qingcai Chen, Kai Chen, Xiaolong Wang, Baohua Wang, Zhe Wang

**Affiliations:** ^1^Intelligent Computing Research Center, Harbin Institute of Technology Shenzhen School, China; ^2^Key Laboratory of Symbolic Computation and Knowledge Engineering of Ministry of Education, Jilin University, China; ^3^College of Mathematics and statistics, Shenzhen University, China.

## Abstract

In this article, an end-to-end system was proposed for the challenge task of disease named entity recognition (DNER) and chemical-induced disease (CID) relation extraction in BioCreative V, where DNER includes disease mention recognition (DMR) and normalization (DN). Evaluation on the challenge corpus showed that our system achieved the highest F1-scores 86.93% on DMR, 84.11% on DN, 43.04% on CID relation extraction, respectively. The F1-score on DMR is higher than our previous one reported by the challenge organizers (86.76%), the highest F1-score of the challenge.

Database URL: http://database.oxfordjournals.org/content/2016/baw077

## Introduction

In recent years, chemicals (or drugs), diseases, and their relations have attracted considerable attention as they play important roles in many areas of biomedical research and healthcare such as biocuration, drug discovery and drug safety surveillance (1). Automatic recognition and normalization of chemical and disease mentions, and automatic extraction of chemical–disease relation (CDR) from literature have become more and more necessary because manual annotation of them is too expensive and insufficient to keep up with the rapid growth of literature.

In last few years, many attempts have been conducted to recognize such entities and to extract such relations automatically using natural language processing methods. However, automatic chemical and disease named entity recognition (DNER) and chemical–disease relation (CDR) extraction remain challenges. The lack of benchmark datasets has seriously limited the development of relative techniques as there is no fair comparison of systems. Through BioCreative V, a challenge task of automatic extraction of mechanistic and biomarker CDRs from the biomedical literature in support of biocuration, new drug discovery and drug safety surveillance was proposed to advance text-mining research on relationship extraction and provide practical benefits to biocuration (2). This task included two subtasks: DNER, including disease mention recognition (DMR) and normalization (DN), and chemical-induced disease (CID) relation extraction. DNER is a preliminary step for CID relation extraction. Beside DNER, chemical named entity recognition (CNER), including chemical mention recognition (CMR) and normalization (CN), is also a preliminary step. Therefore, the related work of CMR and CN was also introduced here.

Most CMR and DMR methods may fall into two categories: rule-based and machine learning-based. The rule-based methods define rules to find CMs and DMs from dictionaries such as Unified Medical Language System (UMLS) (3), ChEBI (4), Medical Subject Headings (MeSH) (5), PubChem (6), DrugBank (7) and Comparative Toxicogenomics Database (CTD) (8) by exact/approximate matching. For example, Vazquez *et al*. (9) proposed a rule-based system to detect drug and chemical compound mentions by building a dictionary about morphological characteristics of the mentions automatically for approximate matching. The machine learning-based methods (10–12) usually regard CMR and DMR as a sequence labeling problem and state-of-the-arts sequence labeling algorithms such as conditional random fields (CRFs) (13) were deployed to them. When ompared with the rule-based methods, the machine learning-based methods usually showed better performance.

Most current CN and DN systems are rule-based (14–16) such as tmChem (17), which defined a series of rules to match items in MeSH and ChEBI for CMs. A small number of normalization systems are based on machine learning methods. For example, DNorm (18), a DN system based on machine learning methods, regarded the normalization problem as an information retrieval problem and used a pairwise learning algorithm to get a ranked list of normalized names.

CID relation extraction is a relation extraction task similar with protein-protein interaction extraction (19) and drug side effect extraction (20). Three types of methods have been proposed for this problem: statistic-based (21), machine learning-based (22) and pattern learning-based (23). The statistic-based methods usually determine CID relations according to co-occurrence frequencies of chemical and disease pairs. The machine learning-based methods regard the CID relation extraction problem as a classification problem and the most popular algorithm used is support vector machines (SVMs). The pattern learning-based methods first extract and rank the syntactical patterns from sentences that contain known pairs from unsupervised corpus, and then discover new pairs based on their associated pattern scores.

In this study, we proposed an end-to-end system for the challenge of automatic extraction of mechanistic and biomarker CDRs, including three subsystems for CMR and DMR, CN and DN and CID relation extraction, respectively. Because CNER (i.e. CMR and CN) is a preliminary step of CID relation extraction, we also presented the performance of our system on CNER although it was not considered for system ranking in the challenge. Evaluation on the corpus of the challenge showed that our system achieved the highest F1-scores of 92.96% on CMR, 86.93% on DMR, 92.19% on CN, 84.11% on DN, 43.04% on CID relation extraction, respectively, higher than the ones reported by the challenge organizers because of post-challenge analysis and improvement.

## Methods

### Dataset

**Figure 1.**
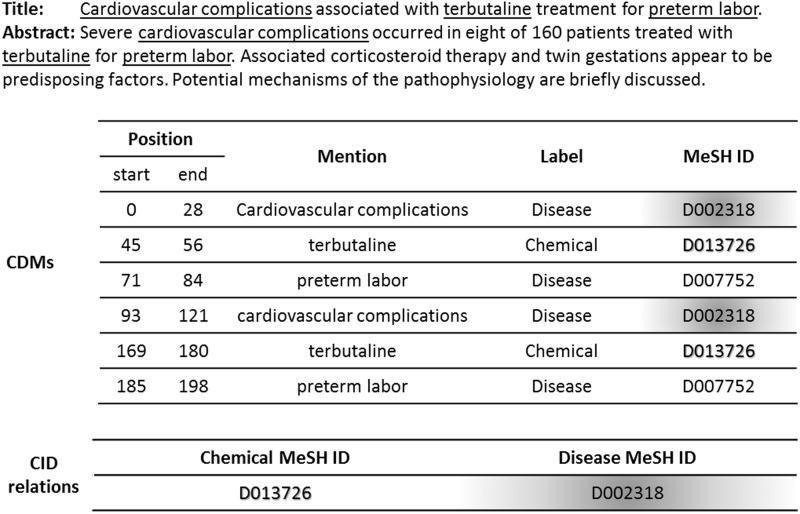
Example of annotated records.

The CDR task organizers of BioCreative V manually annotated 1500 PubMed records, of which 1000 records were used as training and development sets, and the remaining 500 records were used as a test set. Each record consists of two sections: title and abstract, in which not only chemical and disease mentions with MeSH identifiers (IDs), but also CID pairs with relations (i.e. CID relations) were marked up (24). Figure 1 shows an example of annotated records (ID: 7468724), where the consecutive underlined words are CMs and DMs. The statistics of the datasets are listed in Table 1, where ‘T&D’ denotes training and development, ‘DOC’ denotes documents, and ‘#*’ denotes the number of ‘*’.

**Table 1.** Statistics of the dataset for the CDR task of BioCreative V.

Datasets	# DOC	# chemicals		# diseases		# CID relations
		mention	ID	mention	ID	
T&D	1000	10550	2973	8426	3829	2050
Test	500	5385	1435	4424	1988	1066

## Overview of Our System

Our system is an end-to-end system, composed of four modules: a pre-processing module, a module for CMR and DMR, a module for CN and DN, and a CID relation extraction module. Given a PubMed record with title and abstract, the preprocessing module first split it into sentences and tokenized the sentences. Then the CMR and DMR module extracted all CMs and DMs in each sentence. Subsequently, the CN and DN module mapped each extracted mention to a MeSH ID. Finally, the CID relation extraction module found out between which chemicals and diseases there had CID relations. We used the tokenization module of MedEx (25), a specific tool for medical information extraction, for sentence boundary detection and tokenization. The other three modules were presented in detail in the following sections. The system presented here was an improved version of our previous system submitted to the challenge after post-challenge analysis. The main improvement lies in CN and DN.

### Chemical and disease mention recognition (CMR and DMR)

A stacked ensemble system was proposed for chemical and disease mention recognition (CDMR). CDMR was firstly recognized as a sequence labeling problem, and two individual sequence labeling modules: CRFs and structure support vector machines (SSVMs) were deployed. Then, a meta-classifier based on SVMs with a linear kernel (i.e. linear SVMs) was used to check whether a mention recognized by any one of the two previous sequence labeling modules was correct or not. The features used in both CRFs and SSVMs were the same, as shown in Table 2.

**Table 2.** Features used in two individual sequence labeling modules: CRFs and SSVMs.

Feature	Description
Bag-of-words	Unigrams: *w*_0_, *w*_−1_, *w*_1_, *w*_−2_, *w*_0_;
	Bigrams: *w*_−2_*w*_−1,_ *w*_−1,_*w*_0,_ *w*_0_*w*_1,_ *w*_1_*w*_2_;
	Trigrams: *w*_−2_*w*_−1_*w*_0,_ *w*_−1_*w*_0_*w*_1,_ *w*_0_*w*_1_*w*_2_
	*w*_i_ is a token at position relative the current token.
Part-of-speech (POS) tags	Unigrams: *p*_0,_ *p*_−1,_ *p*_1,_ *p*_−2,_ *p*_2_
	Bigrams: *p*_−2_*p*_−1,_ *p*_−1_*p*_0,_ *p*_0_*p*_1,_ *p*_1_*p*_2_;
	Trigrams: *p*_−2_*p*_−1,_*p*_0,_ *p*_−1,_*p*_0,_*p*_1,_ *p*_0,_*p*_1,_*p*_2_
	*p*_i_ is a POS tag at position *i* relative the current token.
Combinations of tokens and POS tags	*w*_−1_*p*_−2,_ *w*_1_*p*_−1,_ *w*_−1_*p*_0,_ *w*_2_*p*_−1,_ *w*_0_*p*_0,_ *w*_0_*p*_1,_ *w*_1_*p*_0,_ *w*_1_*p*_1,_ *w*_1_*p*_2_,
Sentence information	Length of the current sentence; whether there is any bracket unmatched in the current sentence?
Affixes	Prefixes and suffixes of the length from 1 to 5.
Orthographical features	Whether the current word is an upper Caps word? Contains a digit or not? Has uppercase characters inside? Etc.
Word shapes	Any or consecutive uppercase character(s), lowercase character(s), digit (s) and other character(s) in the current word is/are replaced by ‘A’, ‘a’, ‘#’ and ‘-’ respectively.
Section information	Which section the current word belongs to, title or abstract?
Word representation features [5]	Brown clustering (https://github.com/percyliang/brown-cluster);Word2vec (https://code.google.com/p/word2vec/).
Dictionary features	Chemical dictionary: CTD, DrugBank, MeSH, Pharmacogenetics Knowledge Base (PharmGKB) (26), UMLS, and Wikipedia;
	Disease dictionary: CTD, MeSH, UMLS, disease ontology (27), National Drug File Reference Terminology (NDF-RT) (28) and Wikipedia.
Frequency features	Whether the frequency of the current word is higher than a given value (4 in our system) and the inverse document frequency of it is less than another given value (0.1 in our system)?
Character N-grams	Character N-grams (N = 1, 2, …, 4) within the current word.

In the meta-classifier, a variety of features were used to describe the agreement and consistency between the previous modules. Each mention predicted by a sequence labeling module was compared with all other mentions in the same or adjacent position. For each pair of mentions, we extracted the following eight features:

- If the text spans match
- If the text spans partially match (any word overlap)
- If the text spans match and concept types match
- If the text spans partially match and the concept types match
- If the text spans have the same start position
- If the text spans have same end position
- If one text span subsumes the other
- If one text spans are subsumed by the other

Furthermore, given a mention, how many modules predicted it and which module predicted it were also taken into account.

### Chemical and disease normalization (CDN: CN and DN)

**Figure 2.**
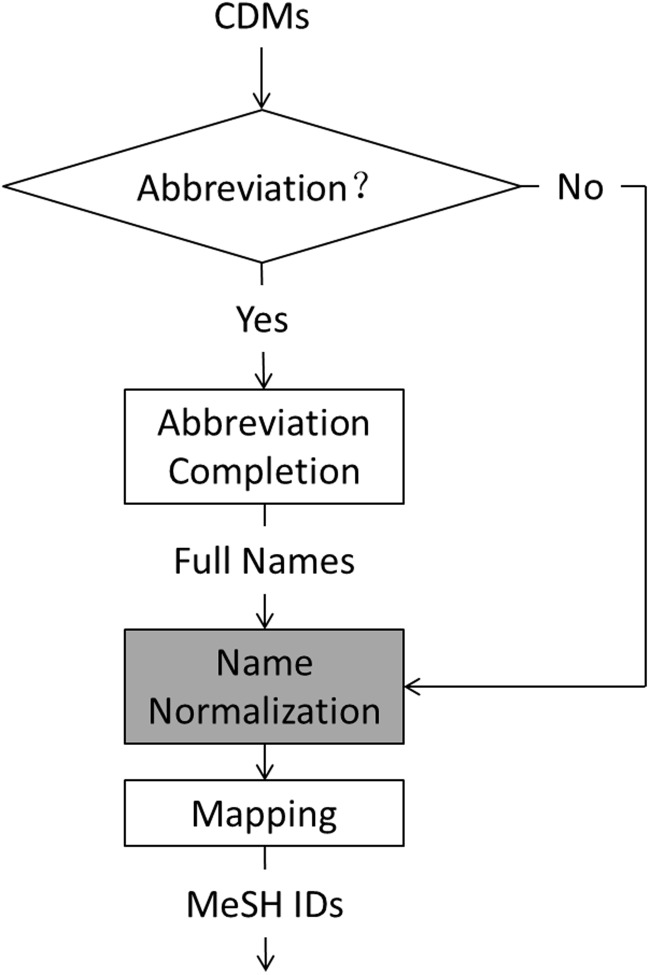
Workflow of our normalization module.

As there were quite a few abbreviations among CMs and DMs such as ‘CAD’, standing for ‘coronary artery disease’ and a CM or DM may have aliases, e.g. ‘ischemic heart disease’, ‘atherosclerotic heart disease’, ‘atherosclerotic cardiovascular disease’ and ‘coronary artery disease’ stand for the same disease, we first completed the abbreviations, then normalized the full names or did not, and finally mapped them to MeSH IDs. Figure 2 shows the workflow of the normalization module in our system, where the “name normalization” in the grey box is an extra option.

We used Ab3P (29) to find full names of abbreviations from records, MeSH WebSearch API (http://eutils.ncbi.nlm.nih.gov/) and Wikipedia API (http://www.wikipedia.com/) for name normalization, and tried different strategies for MeSH ID mapping as follows:

Using dictionary look-up to find MeSH ID.

Combining results of systems using different name normalization module.

**Figure 3.**
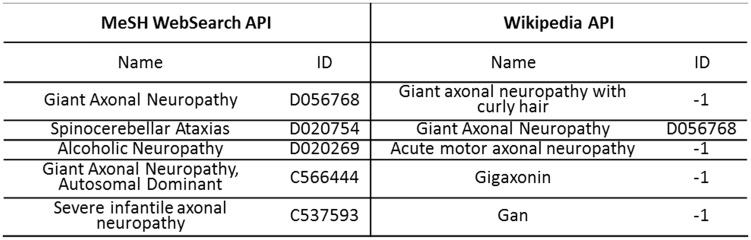
An example of DN for a DM axonal neuropathy using dictionary look-up.

Given a CM or DM, both MeSH WebSearch API and Wikipedia API return a name list. If the first name in the list exactly matches an item in MeSH, the mention is normalized as the item with an ID; otherwise, the mention is discarded. Figure 3 gives an example of DN for a DM ‘axonal neuropathy’ using dictionary look-up. Through MeSH WebSearch API and Wikipedia API, we obtained two name lists of length 5: {‘Giant Axonal Neuropathy’, ‘Spinocerebellar Ataxias’, ‘Alcoholic Neuropathy’, ‘Giant Axonal Neuropathy, Autosomal Dominant’, ‘Severe infantile axonal neuropathy’} and {‘Giant axonal neuropathy with curly hair’, ‘Giant Axonal Neuropathy’, ‘Acute motor axonal neuropathy’, ‘Gigaxonin’, ‘Gan’}, respectively. The first name in the name list returned by MeSH WebSearch API, ‘Giant Axonal Neuropathy’, was found in MeSH with ID D056768, while the first name in the name list returned by Wikipedia API, ‘Giant axonal neuropathy with curly hair’, was not, denoted by ‘ID = −1’.

In addition, a re-ranking system based on SVM-rank was further proposed to combine the results of the above two systems for possible improvement, where all names in the lists as shown in Figure 3 were regarded as candidates for re-ranking. The features used in the system include:

- Bag-of-words of the mention
- Similarity between a candidate and the mention
- Similarity between a candidate and other mentions in the context of the mention
- Whether a candidate generated by MeSH WebSearch API, Wikipedia API or both of them
- Place of a candidate in the ranked list returned by MeSH WebSearch API
- Place of a candidate in the ranked list return by Wikipedia API

Our previous system submitted to the challenge used ‘name normalization’ (see Figure 3).

### CID relation extraction

First, we designed a rule-based filter to generate CID relation candidates, and then built a classifier based on linear SVMs to check whether there was a relation in any candidate. During training, a candidate was assigned as ‘TRUE’ (a postive sample) if it was annotated in the training and development sets, otherwise, it was assigned as ‘FALSE’ (a negative sample). The candidates were generated:

- CID pairs within three sentences. We selected the proper number from {1, 2, 3, 4} according to the recall and precision of the filter on the training and development sets.
- Among the CID pairs with same chemical and drug MeSH IDs generated by step 1, only the pairs between which there have fewest words were kept.
- Among the CID pairs generated by step 2, the pairs between which the number of words was more than a threshold were removed. The threshold was the average number of words between all positive samples generated by step 2 on the training and development sets (i.e. 78).

The features used in the machine learning-based classifier included bag-of-words of both mentions, the number of other mentions between a CID pair, and CDR based on CTD.

## Results

In our experiments, CRFsuite (30), SVM*^hmm^* (31) and liblinear (32) were used as implementations of CRFs, SSVMs and the ensemble meta-classifier for CMR and DMR respectively, SVM^rank^ (33) was used as an implement of SVM-rank for CN and DN, and liblinear was also used as an implement of the SVM classifier for CID relation extraction. To optimize parameters of all subsystems using machine learning methods, we conducted 10-fold cross-validation on the combination of training and development sets. The performance of each subsystem was evaluated by precision (P), recall (R) and F1-score (F1), calculated by the official tool provided by the challenge organizers. As the challenge organizers provided some tools as baseline systems, we compared our system with them. Before introducing results of our system in detail, we should note that all results presented in the following sections may be different from those presented in our previous article (34) as some records failed to be processed because of data transmission problem.

### CDMR: CMR and DMR

Our system achieved F1-scores of 92.96 and 86.93% on CMR and DMR, respectively, as shown in Table 3, where the F1-score on DMR is a little better than that of our previous system submitted to the challenge (86.76%), the highest F1-score of the challenge (34), due to data transmission problem. When compared with the baseline systems: tmChem and DNorm, provided by the organizers, our system showed much better performance. It outperformed tmChem by 4.36% on CMR and DNorm by 6.69% on DMR, respectively. When compared with CRFs and SSVMs, the stacked ensemble method performed slightly better, due to significantly higher precision. For example, on DMR, the stacked ensemble method outperformed SSVMs by 0.05% in F1-score because of much higher precision (88.68% vs 87.74%). Among CRFs and SSVMs, SSVMs achieved higher F1-score, mainly due to higher recall. SSVMs outperformed CRFs by 0.6 and 0.1% in F1-score on CMR and DMR, respectively. The differences between recalls were 0.95 and 0.82%.

**Table 3.** Results of our system on CMR and DMR (%).

Method	Chemical			Disease		
	P	R	F1	P	R	F1
*DNorm*	NA	NA	NA	81.62	78.91	80.24
*tmChem*	93.08	84.53	88.60	NA	NA	NA
*CRFs*	94.25	90.44	92.30	88.37	85.23	86.78
*SSVMs*	94.58	91.35	92.93	87.74	86.05	86.88
*Stacked ensemble*	95.05	90.96	92.96	88.68	85.23	86.93

### CDN: CN and DN

The direct dictionary look-up system (without using name normalization mentioned in Figure 2) outperformed the two dictionary look-up systems using name normalization by at least 2.51% in F1-score on CN and 3.77% in F1-score on DN. Among the two dictionary look-up systems using name normalization, the system using Wikipedia API for name normalization was a better one. The re-ranking system taking the outputs of the two dictionary look-up systems using name normalization as input did not bring any improvement. The highest F1-scores of our system on CN and DN were 92.68 and 84.11%, respectively, much higher than four baseline systems: ‘dictionary look-up’ that directly looked up MeSH using DMs from CTD, ‘tmChem’, ‘DNorm’ and ‘DNorm*’ that adopted DMs from our DMR system as input of DNorm for DN, as shown in Table 4. The smallest F1 differences between the baseline systems and our system on CN and DN were 4.67 and 2.82%, respectively. The reason why our previous system submitted to challenge used name normalization module (see Figure 2) and only achieved a highest F1-score of 67.82%, much lower than that presented here (78.24%), is just because the name lists returned by MeSH WebSearch API and Wikipedia API were sometimes empty within a limited time for communication between our system and the challenge server.

**Table 4.** Results of our system on CN and DN (%).

Method	Chemical			Disease		
	P	R	F1	P	R	F1
*dictionary look-up*	NA	NA	NA	42.71	67.46	52.30
*tmChem*	95.02	81.11	87.52	NA	NA	NA
*DNorm*	NA	NA	NA	81.15	80.13	80.64
DNorm*	NA	NA	NA	81.25	81.33	81.29
*MeSH*	87.15	90.73	88.90	77.89	80.43	79.14
*Wikipedia*	87.95	91.43	89.68	78.62	82.14	80.34
*Re-ranking*	87.83	90.03	88.92	78.36	78.11	78.24
*Mapping directly*	93.48	90.94	92.19	88.64	80.03	84.11

### CID relation extraction

Table 5 showed the performance of our system when using the output of different CN and DN systems (as mentioned earlier) as input. The system taking the output of the “Mapping directly” CN and DN system (see Table 4) achieved the highest F1-score of 43.04%, outperforming the systems taking the other three CN and DN systems: ‘MeSH WebSearch’, ‘Wikipedia’ and ‘Re-ranking’ (see Table 4) as input by 4.97, 3.77 and 5.87%, respectively. When compared with two baseline systems: (i) ‘Co-occurrence’ that took output of both tmChem and DNorm as input, and determined CID relations according to the frequency of CID pairs; (ii) ‘Co-occurrence*’ that took output of the ‘Mapping directly’ CN and DN system (see Table 4) as input, and determined CID relations according to the frequency of CID pairs, our system showed much better performance. The F1-score difference between the baseline systems and our system achieved 13.15% (29.89 vs 43.04%). Among the two baseline systems, ‘Co-occurrence*’ was a much better one, indicating that the ‘Mapping directly’ CN and DN system is much better than tmChem and DNorm again. When compared with our previous systems submitted to the challenge that took the outputs of the three CN and DN systems: ‘MeSH WebSearch’, ‘Wikipedia’ and ‘Re-ranking’ as input, the current systems also achieved much better performance (the previous highest F1-score of 41.26% (34) vs the current highest F1-score of 42.65%) because that there was no data transmission problem in the current systems.

**Table 5.** Results of our system on the CID relation subtask (%).

Method	P	R	F1
*Co-occurrence*	16.43	76.45	27.05
Co-occurrence*	18.51	77.65	29.89
*MeSH WebSearch*	53.82	34.33	41.92
*Wikipedia*	54.61	34.99	42.65
*Re-ranking*	55.83	34.15	42.37
*Mapping directly*	57.93	34.24	43.04

## Discussion

An end-to-end machine learning-based system was proposed for the CDR extraction challenge of BioCreative V, composed of three subsystems: CMR and DMR, CN and DN and CID relation extraction. For each subsystem, we investigated the performance of different methods.

On CMR and DMR, similar with previous studies (34) on other named entity recognition problems, the stacked ensemble method outperformed than CRFs and SSVMs (see Table 3). The main reason lies in that the stacked ensemble method is able to make a good choice when there are conflicts between CRFs and SSVMs, resulting in higher precision. For example, given a tokenized sentence fragment ‘We have described a patient with severe rheumatoid arthritis and a history of mefenamic acid nephropathy in whom hyperkalemia and inappropriate hypoaldosteronism ……’ with one CM (i.e. ‘mefenamic acid’) and three DMs (i.e. ‘rheumatoid arthritis’, ‘hyperkalemia’ and ‘hypoaldosteronism’), all CMs and DMs were correctly recognized by CRFs and SSVMs at the same time except the DM ‘hypoaldosteronism’, which was correctly recognized by CRFs, but wrongly recognized as ‘inappropriate hypoaldosteronism’ by SSVMs. The stacked ensemble method chooses the correct DM. Nevertheless, there were still a large number of errors in our system, such as abbreviations that could not be recognized. In future work, we will try to develop a post-processing to further handle boundary errors and abbreviations.

On CN and DN, by checking errors of our dictionary look-up systems using two name normalization tools, we found that a large number of errors were caused by name normalization (see Figure 2) such as ‘neuroneal lost’ wrongly normalized as ‘Frontotemporal Lobar Degeneration’ by MeSH WebSearch API and ‘amphotericin’ wrongly normalized by Wikipedia API. This may be also the main reason why the re-ranking method performed worse than the two individual dictionary look-up system using name normalization. When compared with the best DN system of the challenge, which was also a dictionary look-up system (35), our system is still inferior. The main difference lies in that the best system of the challenge considered more accurate domain dictionaries and rules for name normalization.

It is easy to understand that the CID extraction system taking the output of the ‘Mapping directly’ CN and DN system (see Table 5) outperformed the other systems for comparison since the ‘Mapping directly’ CN and DN system was better than other CN and DN systems as shown in Table 4. When compared with the best CID relation extraction system of the challenge (36), our system is still not good enough. The main factors affecting the performance of our CID extraction systems include: (i) whether the candidate filter is good enough? The more positive samples and less negative samples in candidates, the better the candidate filter is; (ii) which machine learning algorithm and which features you use? The numbers of positive and negative samples in candidates generated by the filter in our CID extraction system on the training and development sets are 1530 and 5079. It means that the recall of the filter is 74.63% (1530/2050, where 2050 is the total number of CID relations in the training and development sets as shown in Table 1), which is a little low, and the precision of the filter is 23.15%, which is imbalanced. During the limited time of the challenge, we only developed a simple CID extraction system with limited features. For further improvement, we will try to other strategies to generate better candidates with higher recall and precision, and try other machine learning algorithms and much richer features such as features generated by deep learning algorithms.

## Conclusion

In this article, we introduced an end-to-end system for the challenge of automatic extraction of mechanistic and biomarker CDRs in BioCreative V, which consists of three subsystems corresponding to CMR and DMR, CN and DN and CID relation extraction. Ensemble learning methods slightly outperformed two individual state-of-the-art machine learning methods (i.e. CRFs and SSVMs) on CMR and DMR when they were regarded as sequence labeling problems, and achieved the best performance on DMR as far as we known. On CN and DN, a good name normalization module is a key point of dictionary look-up methods. The CID relation extraction remains a challenge.
